# Clinical staging for eating disorders: A scoping review and update

**DOI:** 10.1192/j.eurpsy.2026.12241

**Published:** 2026-07-21

**Authors:** Lucy Hyam, Lucy Gallagher, Carina Kuehne, Melissa-Claire Daugelat, Melissa J. Pehlivan, Regan Mills, Karina L. Allen, Ulrike Schmidt

**Affiliations:** 1 Centre for Research in Eating and Weight Disorders, Institute of Psychiatry, Psychology and Neuroscience, King’s College London, London, UK; 2 Department of Psychosomatic Medicine and Psychotherapy, Medical University Hospital Tübingen, Tübingen, Germany; 3 Centre of Excellence for Eating Disorders Tübingen (KOMET), Eberhard Karls University Tübingen, Tübingen, Germany; 4 InsideOut Institute for Eating Disorders, The University of Sydney, Australia; 5 Department of Psychology, University of Bath, UK; 6 Eating Disorders Outpatients Service, South London and Maudsley NHS Foundation Trust, London, United Kingdom

**Keywords:** diagnosis, eating disorders, treatment, anorexia, bulimia, binge eating disorder

## Abstract

**Background:**

There has been growing interest in developing a clinical staging model for eating disorders (EDs). The aim of this scoping review is to investigate the extent, range, and nature of research that can inform clinical staging approaches for EDs.

**Methods:**

This review was conducted following the Joanna Briggs Institute methodology for scoping reviews and the PRISMA extension for Scoping Reviews. A search of PubMed, PsycINFO, MEDLINE and Web of Science databases was conducted. Sixty studies were included for review, and data were extracted according to revised pre-published criteria. Studies were summarized using narrative synthesis.

**Results:**

Thirty-six of the included manuscripts were quantitative, 18 were qualitative, and six were conceptual/review papers. The included studies primarily focused on anorexia nervosa in white, female samples and compared only two stages of illness, equating to “early” and “longstanding” or “severe and enduring” EDs. “Middle” stages or transitions between stages were not well-defined. Stages have mostly been conceptualized using duration of illness or other behavioral or clinical parameters, with few (neuro) biological studies identified. Factors that may modify illness stage have not yet been integrated into ED staging literature.

**Conclusions:**

There is growing consensus that early- and later-stage EDs have distinct characteristics and treatment requirements; however, these distinctions require clearer definition. Recommendations for future research are outlined, emphasizing integration of psychosocial and biological research, focused investigation of illness phases outside of early and longstanding phases, and evaluation of personalized, stage-based treatments. A tentative staging model for EDs is proposed.

## Introduction

Eating disorders (EDs) are common and serious mental health conditions. Their main treatments are psychological or nutritional, with medications playing only a limited role [1]. The etiology of EDs is biopsychosocial, with large genome-wide association studies pointing toward shared and unique metabo-psychiatric origins across ED subtypes [2]. Even with the best available treatments, only around 50% of those with EDs recover, and 20%–30% develop a chronic and/or disabling illness [1].

It is unclear how or why some people develop more severe and persistent EDs. Neuroimaging and behavioral studies highlight the importance of habit formation (the process by which contextual stimuli come to drive behavior independently of reward [3]) in the development and persistence of maladaptive behaviors in anorexia nervosa (AN) [4] and binge spectrum disorders (e.g., bulimia nervosa [BN], binge eating disorder [BED]) [3]. Habit strength also increases with longer illness duration [5]. Network analyses demonstrate a shift in symptom profiles over time, with cognitive symptoms (over-valuation of weight, shape, and appearance) central in shorter-duration illness (<3 years) and behavioral symptoms (e.g., dietary restriction, compulsive exercise) central in longer-duration illness (≥10 years) [6, 7]. Although such findings allude to the processes that may shape different ED trajectories, traditional illness and treatment models struggle to capture these fluid and varied illness presentations in mental health [8].

Clinical staging offers a framework toward better delineating illness progression and severity, defining a continuum of disorder progression from asymptomatic or at-risk states, into early forms of illness, and late-stage, severe, or more persistent illness [9, 10]. Originating in oncology [11, 12], established staging models of illness have advanced prevention and early intervention. By clearly defining emerging and early forms of illness and intervening at these stages, progression to more established forms of illness can be delayed or averted [13].

In psychiatric research, disorder-specific and transdiagnostic staging models have been proposed to better reflect the spectrum, progression, and heterogeneity of mental illness [8]. Early work focused primarily on psychotic and severe mood disorders, defining stages based on progression status, likely population, indicative biological/endophenotypic biomarkers, and potential interventions [14].

Research on staging in EDs is still limited [9]. A recent systematic scoping review sought documents on explicit staging models or empirical evidence testing them, identifying nine staging models, which primarily focused on AN [15]. Models varied in their definitions and number of stages, with no consistent criteria, only two had been empirically tested, and evidence for stage-targeted treatments was lacking. Models generally followed a continuum from early/at-risk stages through acute illness to chronic presentations. Remission, relapse, and recovery stages were limited or poorly defined. Further empirical testing, application to broader ED populations, and integration of biomarkers were identified as key research priorities.

Further refinements within the wider staging literature include adding progression (“P” factors) and extension (“E” factors) stage modifiers to better capture individual differences in illness course and guide treatment selection [8]. P factors describe the development and changing of the core disorder over time (e.g., at-risk to full symptoms). E factors refer to additional related symptoms or conditions (e.g., comorbidities, physical health problems, or metabolic factors). P factors map out the longer-term prognosis and likelihood of progression, whereas E factors reflect the added complexity of an individual presentation. These have been applied primarily in psychotic and mood disorders and, to the best of our knowledge, have not yet been integrated within ED staging models.

This scoping review aims to build upon recent work [9, 15] by incorporating a broader range of evidence beyond explicit staging models, such as qualitative studies and research on clinical, psychosocial, and biological factors that may inform illness progression and potential stage modifiers. Other feeding disorders, such as Avoidant/Restrictive Food Intake Disorder (ARFID) are also considered to assess if staging literature is applied to the full spectrum of EDs.

Our research questions are:

1. What is the scope, range, and nature of research investigating illness staging paradigms for EDs?
2. What evidence is available for specific illness stages, including high-risk/prodromal stage, early stage, and late-stage illness across different EDs?
3. Which different clinical, biological, and neuroscientific domains of assessment/functioning does this research cover?
4. What evidence is there that demonstrates the implications of different illness stages for treatment and outcomes across the EDs?

## Methods

A scoping review design was adopted, given our exploratory research aims. Further, Tomba et al.’s recent review [15] on staging models included only 11 studies; thus, a broader focus was considered necessary to map the full range of potentially informative literature. This review followed the Joanna Briggs Institute (JBI) methodology for scoping reviews and the PRISMA extension for Scoping Reviews [16, 17]. Significant modifications of the protocol [18] were required to reduce the number of irrelevant studies identified (see Supplementary Material). The revised inclusion criteria are listed in Table 1.

**Table 1.** Inclusion criteria Table 1. long description.

Category	Details
Participants	Clinical and non-clinical samples with disordered eating or diagnosed ED (including AN, BN, BED, OSFED, or ARFID).
Concept	Studies that compare clearly defined stages or phases of ED (e.g., high-risk, early, or late-stage), or Propose a complete staging model or framework, or Compare groups based on illness duration (a common proxy for conceptualizing illness stage or severity), or Examine clinical, psychosocial, or biological variables in relation to illness progression (e.g., associations between duration of illness and severity markers) that are relevant to informing clinical staging.
Context	Any healthcare or community setting, with no geographical restrictions.
Evidence types	All types of documents and study designs.
Language	Only English language publications.

Abbreviations: AN, anorexia nervosa; ARFID, avoidant restrictive food intake disorder; BED, binge eating disorder; BN, bulimia nervosa; ED, eating disorder; OSFED, other specified feeding or eating disorder.

### Search strategy

Searches were conducted on PubMed, PsycINFO, MEDLINE, and Web of Science. After discussion and testing the search terms, it was decided to remove “severity,” “transdiagnostic,” “biomarker,” and “presymptomatic” to refine the search. Exact search terms are available in supplementary Table S3. Briefly, variations of the terms “eating disorders,” “clinical staging,” “progression,” “illness stage,” and “persistent” were used.

In total, three searches were completed. The first search was completed on 5 January 2024 and the second on 10 June 2025. A top-up search was conducted on 10 January 2026. Identified studies were imported into Endnote citation management software [19] and duplicates were removed. All screening was completed in Rayyan screening software [20]. Six authors screened titles, abstracts, and full texts; subsequent searches were screened by new authors but according to the same criteria. Disagreements were brought to the senior authors (U.S. and K.A.) for a decision.

Given the scoping nature of the review and the substantial overlap observed between databases in the initial search, the top-up search was conducted on PubMed and Embase only.

### Data charting

A charting form was adapted from the template from the JBI manual, with some changes made to the protocol (detailed in Supplementary Table s1). Data extraction was completed by three authors, and a random selection (*n* = 11) of the studies was cross-checked by a separate author for quality checking. Each source was appraised using the JBI Levels of Evidence for effectiveness, prognosis, and meaningfulness by two authors [21].

A narrative synthesis of the text is provided to address the research questions, with findings organized by study design, clinical domain, and implications for staging.

## Results

### Identification of evidence

The initial searches identified 18,907 records across PubMed, Medline, and PsycINFO via OVID, and Web of Science. After removal of 10,918 duplicates, 7,989 records underwent title and abstract screening, with 6,883 excluded. Full-text screening was conducted for 240 articles, of which 195 were excluded, and 55 were included.

The top-up search retrieved 775 records (397 PubMed; 378 Embase). After removing 169 duplicates, 606 records proceeded to title and abstract screening. Seven studies progressed to full-text screening, and a total of five studies were included from this search, resulting in a total of 60 studies for inclusion.

**Figure 1.**
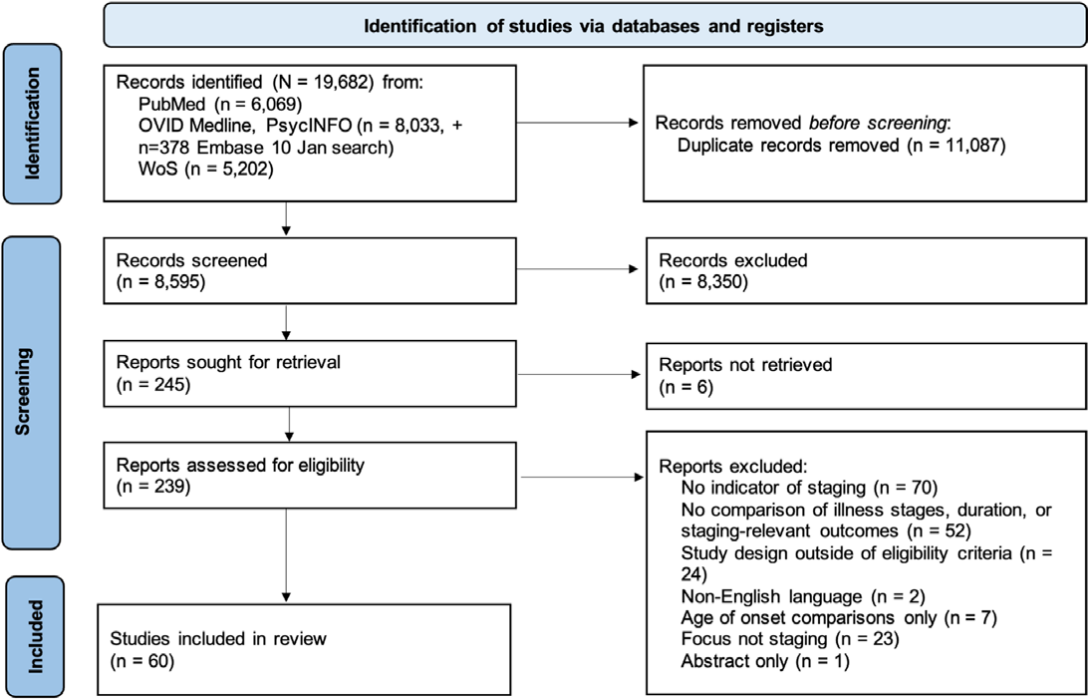
Flow diagram of the review search. Figure 1. long description.

The flow of screening and selection of records is shown in Figure 1. Condensed tables detailing the included studies and extracted data are available in Supplementary Material S1 (Supplementary Tables S4–S6). The full table of extracted data (spanning 28 A3 pages) is available from the authors upon request.

### Features of included studies

**Figure 2.**
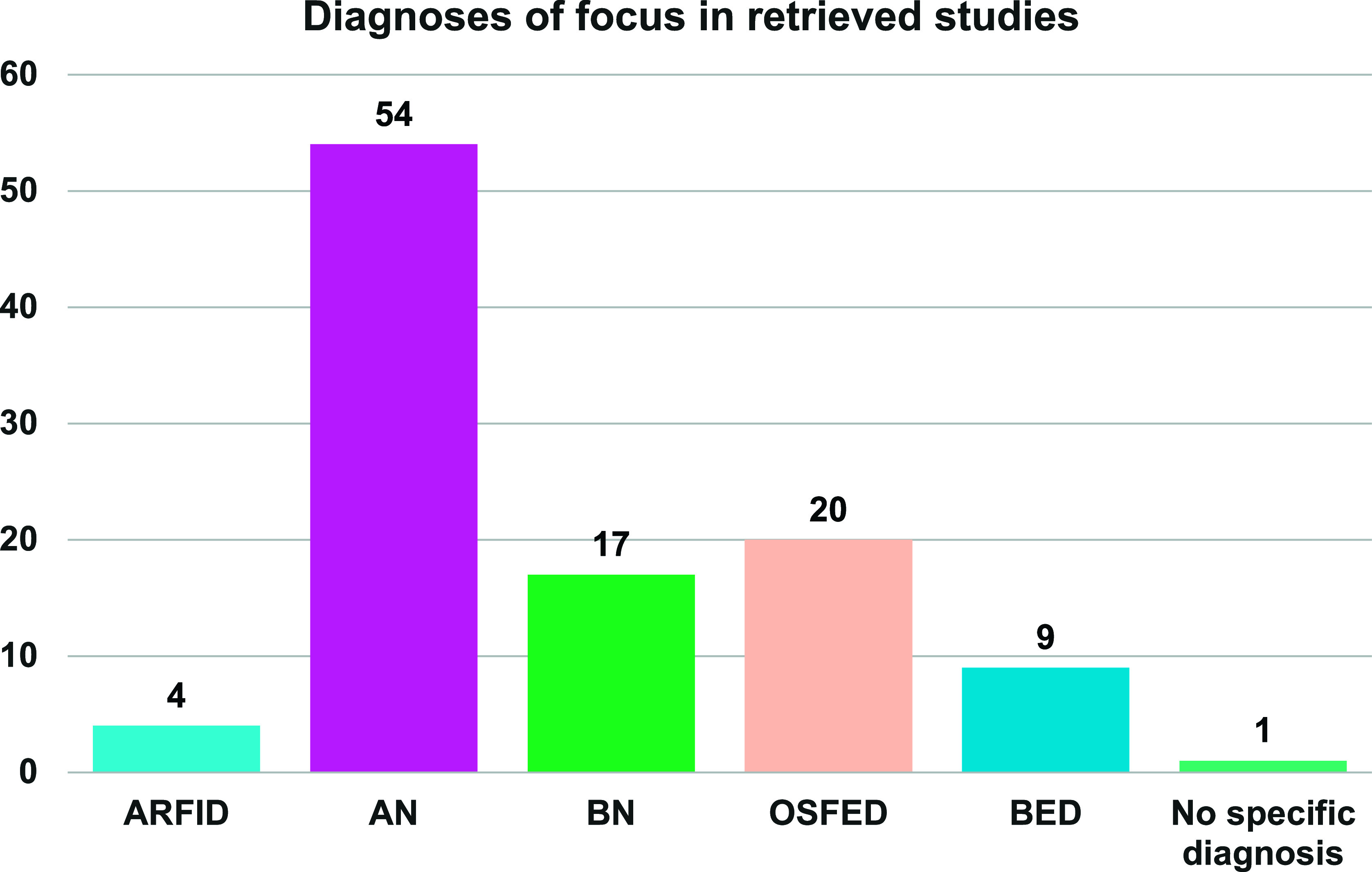
Cumulative frequency bar charts of diagnoses included in papers. *Note*: Papers focusing on multiple EDs were counted in each relevant diagnostic category rather than as a single combined group; therefore, totals exceed the number of included studies. AN, Anorexia nervosa; ARFID, avoidant/restrictive food intake disorder; BED, binge eating disorder; BN, bulimia nervosa; OSFED, other specified feeding or eating disorder.

Of the 60 included sources, 36 were quantitative studies, 18 were qualitative studies, and six were conceptual/review papers. These mostly originated from European countries (*n* = 30; UK: *n* = 8; Italy: *n* = 7; Germany: *n* = 3; France: *n* = 3; Portugal: *n* = 2; Sweden: *n* = 2; Spain: *n* = 1; Czech Republic: *n* = 1; Netherlands *n* = 1; Norway: *n* = 1; Ireland: *n* = 1), followed by the USA (*n* = 14), Australia (*n* = 11), Canada (*n* = 1), and Chile (*n* = 1). Three papers had samples or authors from multiple countries. No manuscripts were retrieved from Africa or Asia. Study design and diagnoses examined are summarized in Table 2 and Figure 2.

**Table 2.** Study designs, qualitative methods, and analytic approaches represented in the included literature Table 2. long description.

Category	Subtype	Count
Quantitative study designs	Clinical trial	1
	Prospective or retrospective cohort cohort	2
	Cross-sectional	15
	Longitudinal	16
	RCT	2
*n*		**36**
Conceptual/review designs	Systematic review	3
	Consensus methods	1
	Theoretical review	2
*n*		**6**
Qualitative methods	Meta-synthesis	3
	Interviews/focus groups	11
	Photovoice	1
	Autoethnography	1
	Case study	2
*n*		**18**
Qualitative analytic approaches	Narrative approach	2
	Thematic analysis	9
	Grounded theory	1
	Phenomenological analysis	3
	Descriptive analysis	1
	Meta-ethnography	2
*n*		**18**

Abbreviation: RCT, randomized controlled trial.

Studies were predominantly clinical or behavioral in focus, examining psychological symptom severity, treatment response, and BMI change [22–51]. A smaller number examined neurobiological domains, such as a dopamine-based staging model [52], bone health, cardiovascular health, and gut microbiome structure and metabolic signatures [53–58]. Some studies investigated brain structure and function [47, 52, 59–62]. Qualitative studies focused on psychosocial domains such as personality, identity, autonomy, and trauma [63–81].

Demographic information is not reported for the conceptual/review papers. Across 54 qualitative and quantitative studies, age was reported in 52 (96.3%), gender in 49 (90.7%), and ethnicity in 18 (33.3%) studies.

### Staging in quantitative papers

A variety of methods were used to operationalize ED “stages” (see Table 3). A minority of studies looked dimensionally, examining symptom severity continuously from subsyndromal to full-symptom EDs [23, 46]. Duration of illness (DOI) was used as the primary marker of stage, with several studies relying on DOI alone to distinguish stages/phases [25–27, 30, 34, 35, 37, 38, 40, 42, 43, 50, 53, 55, 58, 60]. A handful of studies added additional clinical metrics to DOI-based stages. These included measures of ED-related psychosocial impairment, depression, anxiety, and stress [24, 41, 51].

**Table 3.** Quantitative studies – stage conceptualizations Table 3. long description.

Category	Staging criteria	Details (number of groups, stage label)
1. Clinical Scores	Symptom or diagnostic status	Subthreshold – full ED using symptomatic (Child EDE-Q [82] and EDE-Q [83]) assessments [23] Prodromal – full ED onset using diagnostic (EDDI [84]) interviews [46] Recovery, remission, relapse (LIFE [85] and COST Action B6 criteria [86]) [44]
2. DOI-only stages	DOI grouping only	Two groups: <3 years (short duration), >3 years (long duration) [40] <3 years, >3 years [30] <3 years (first episode), >7 years (persistent) [55] <3 years (acute), >7 years (SE-ED) [38] <3 years (early stage), >7 years (longstanding AN) [50] <3 years (acute), >7 years (SEAN) [58] <6, >6 years [34] <7 years (non-SE-AN), >7 years (SE-AN) [27] <7 years (short-term), >7 years (long-term) [43] <10 years (shorter ED), >10 years (longstanding ED) [26] Three groups: Onset, established (mean DOI 2.5 years), recovered [53] <6 months, 7–12 months, >1 year [35] <1 year, 1–2 years, >2 years [25] <3 years (short), 3–<7 years (medium), >7 years (long) [37] 3, 7, or 11 years [42] <5 years (acute), >5 years (chronic), >5 years long-term recovered [60]
DOI + another variable	DOI combined with other clinical variables	Two groups: DOI <3 years (early AN), >7 years (SE-AN) + DASS ≥60 [24] DOI <7 years versus ≥7 years + CIA ≥16 [41] Three groups: EDE-Q severity 2.3–3.4 (at risk), ≥3.5 (acute), DOI <7 years (clinical group) [51] Four groups: Operationalizing multidimensional severity scores into stages [36] Stage 1: Mild AN (<1 month–<6 months) Stage 2: Moderate AN (>6 months–<2 years) Stage 3: Severe AN (2 years–5 years) Stage 4: Extreme AN (>5 years)
Single stage + predictors	One stage assessed with other indicators	SE-AN classification tested via severity and chronicity indicators [48]

Abbreviations: AN, Anorexia nervosa; CIA, Clinical Impairment Assessment questionnaire [87]; COST, European Cooperation in Science and Technology; DASS, Depression Anxiety Stress Scale [88]; DOI, duration of illness; ED, eating disorder; EDDI, Eating Disorder Diagnostic Interview [84]; EDE-Q, Eating Disorder Examination Questionnaire [83]; LIFE, Longitudinal Interval Follow-up Evaluation; SE-AN, Severe and enduring anorexia nervosa; SE-ED, severe and enduring eating disorder.

One study empirically tested a multi-component AN staging model incorporating measures of motivation, weight, obsessionality, bulimic behaviors, and “acute issues” [36]. One study focused on a single “severe and enduring AN” (SE-AN) stage only, but examined associations among several indicators (DOI, treatment history, BMI, binge eating, purging, quality of life) to investigate whether distinct subgroups or dimensions emerged within this stage [48]. Another examined stages of partial remission and subsequent relapse in AN and BN, identifying disorder-specific predictors and high-risk periods to inform illness progression [44].

Definitions of stages varied widely. Some described only two stages, usually an “early stage” and a “severe and enduring” stage, using DOI thresholds ranging from under a year to more than a decade. Others outlined several stages, from acute or recent-onset illness through to more established or chronic presentations. A small number also included a recovery stage [53, 60].

Finally, several studies did not explicitly investigate specific illness stages, but explored the relationship between DOI and various clinical outcomes (see Table 4). While these studies did not evaluate defined stages, they were included as DOI is often used to approximate illness stage within the literature.

**Table 4.** Quantitative studies – duration of illness associations Table 4. long description.

Category	Summary
DOI associations with outcomes	Analyzed across pre-defined outcome groups or examined as a continuous variable with outcomes:
	Compared across three outcome groups defined by dietary restriction changes [22]
	Compared across four outcome groups defined by BMI trajectory [31]
	Four outcome groups in longstanding ED based on prior treatment response [32] Associated with four outcome groups, with personality and psychopathology [39] Associated with two outcome groups [61]
	Associated with ED severity and treatment-seeking behavior [29]
	Associated with outcomes and explored for potential staging [33]
	Associated with myocardial mechanics [54] Symptomatic assessments used to identify relapse predictors [44]
	DOI and amenorrhea duration associated with bone mineral density and hip strength [56]
	Associated with bone mineral density [57]
	Associated with insular cortex volume [62] Personality assessed across four long-term outcome groups defined by clinical status [79]

Abbreviations: BMI, body mass index; DOI, duration of illness; ED, eating disorder.

### Staging in qualitative studies

Table 5 summarizes the stages examined and definitions used in qualitative studies. These studies mostly focused on a later stage of AN, with only two studies examining early ED stages [73, 81]. A minority of studies took different approaches. One compared subclinical versus full-threshold BN using symptom scores [76], and another explored how individuals with high versus low polygenic risk for AN describe differences in illness onset, course, symptom experience, and recovery [80].

**Table 5.** Qualitative studies – stage conceptualizations Table 5. long description.

Category	Staging criteria	Details (number of groups, stage label)
1. Clinical Scores	Symptom, diagnostic, or polygenic status	Subclinical to full-threshold BN using symptomatic assessments (EDI–3, SCID-IV) (1) [76] High versus low polygenic risk score for AN and differences in illness trajectories (1) [80]
2. DOI	DOI grouping only	One group: ≤3 years (early-stage AN) (1) [73] <3 years (early-stage EDs) (1) [81] >3 years (longstanding AN) (2) [70, 71] >3 years (longstanding EDs) (1) [81] ≥5 years (longstanding BN) (1) [74] ≥7 years (longstanding AN) (4) [63–65] ≥10 years (longstanding AN) (1) [69] 14–28 years (longstanding AN) (range) (1) [77] 18 years (longstanding AN) (1) (single case) [72] >20 years (longstanding AN) (1) [75] 25 years (longstanding AN) (1) (single case) [68] 6.3–22.4 years (average, all EDs) (1) [66]
DOI + other criteria		One group: ≥7 years + ≥1 inpatient treatment + severe impairment across two life domains [67]
3. Named stage		One group: Criteria not specified (all EDs) (1) [78]

Abbreviations: AN, anorexia nervosa; BN, bulimia nervosa; DOI, duration of illness, EDI-3, Eating Disorders Inventory-3 [89]; EDs, eating disorders; SCID-IV, Structured Clinical Interview for DSM-IV [90].

The terminology/labels used for longstanding EDs varied, with the majority referring to severe and enduring AN (“SE-AN”) [67, 70–72, 77] or “longstanding AN” [63–65, 68]. Other studies used terms such as “chronic AN” [69], “longstanding and severe ED” [66] “SE-ED” [78], “enduring ED” [81], “SEED-AN” [75], or “SEED-BN” [74].

### Staging in review/conceptual papers

For the six review/conceptual papers, a range of staging approaches were proposed. Some emphasized stages based on specific mechanisms involved in the progression of an ED. For example, Beeler and Burghardt proposed two stages of “development” and “entrenchment” in AN, focused on dopaminergic plasticity and collapse; early restriction and exercise initially elevate dopamine, reinforcing learning and the development of AN behaviors [52]. With continued restriction, the system collapses, reducing plasticity and leading to increasingly inflexible and compulsive behaviors.

Bodell and Racine focus on reward dynamics in BED, proposing a three-stage model from risk, to recent-onset illness, to established illness [59]. At-risk individuals show heightened wanting and liking; recent-onset illness shows food-specific reward tuning; and in later stages, wanting remains high but liking drops, driving binge eating without satisfaction. Rizk et al. introduce a five-stage model of problematic physical activity in AN (the inappropriate intensity, frequency, or duration of activity, or a problematic relationship to activity), highlighting voluntary and biologically driven components of excessive exercise and its association with poorer outcomes [49].

Other models outlined broader trajectories. Cosci and Fava, and Treasure et al., outlined four-stage models for AN and BN and across EDs, respectively [28, 47]. These trace the linear progression of EDs from prodromal features (e.g., “safe foods,” emerging mood and anxiety symptoms), to acute manifestations (e.g., restriction, compulsive exercise, binge-purge cycles), to residual symptoms, and finally to chronic phases characterized by entrenched behaviors, medical complications, and psychiatric comorbidity. Steinglass et al. outline a five-stage model of AN based on expert consensus, defining stages from subsyndromal to full syndrome, persistent illness, and stages of partial or full remission [45].

### Synthesis and application to staging

Across quantitative studies, longer DOI in AN was associated with poorer psychological, work, and social well-being outcomes [24, 41]. Across studies using a range of ED samples, longer DOI was sometimes associated with poorer treatment outcomes [22, 25, 30, 34] and with delays in accessing specialized treatment [29]. However, other studies in AN or underweight OSFED samples reported mixed or no clear differences in treatment response or dropout for different DOI groups [26, 27, 42, 43]. For example, effects were sometimes only present alongside other factors such as higher age [30] or comorbid depression [34].

Effects on biological markers also varied; longer DOI in AN was associated with worse myocardial strain, suggesting compromised heart function [54], and altered insular cortex structure [62]. Findings relating to bone health were mixed. In AN, bone density loss increased with longer DOI, but improved with recovery [53], whereas another study demonstrated increased fracture risk compared to healthy controls for women with both first-episode and persistent AN [55]. In contrast, a study in males across AN, subthreshold AN, atypical AN, and ARFID reported lower bone mineral density associated with longer DOI, reduced muscle mass, and vitamin D deficiency [57].

One study used mixture modeling to investigate whether longstanding AN may be better represented by a hybrid of severity groups and continuous dimensions rather than DOI alone. The authors found no evidence for discrete classification based on chronicity; instead, a hybrid model comprising two severity groups (higher versus lower) and three continuous dimensions reflecting ED behaviors, quality of life, and illness burden, with DOI serving as a continuous indicator of chronicity [48].

Qualitative studies mostly explored the lived experience of long-duration EDs. Longstanding AN was associated with ambivalence, diminished motivation, and loss of confidence in treatment, although individuals still hoped for meaningful improvement [67, 69, 74, 75]. Participants across studies described unmet treatment needs or harmful experiences of treatment [50, 63, 64, 67, 68, 71]. Longstanding EDs were often associated with past trauma or abuse [63, 64, 66, 68] and longstanding AN was frequently linked to identity and serving functional roles such as regulating emotions or relationships [64, 66–69, 72]. One study found that individuals at higher polygenic risk described a more persistent, internally, identity-driven illness course [80].

Only one study investigated longstanding BN, demonstrating enduring physical problems, such as dental and gastrointestinal symptoms, difficulties in relationships, and reduced confidence in treatment [74]. Another study investigating subclinical to full-threshold BN found that while core factors such as body image concerns and fear of rejection were shared across diagnostic status, they varied in intensity and those with full-threshold BN reported greater loneliness, distress, and more intense binge-purge cycles [76].

## Discussion

This scoping review aimed to map literature informative for clinical staging in EDs. Studies generally covered three broad stages: a high-risk/prodromal stage of early symptoms or risk factors; an early stage of recent-onset illness and response to treatment (commonly defined as within <3 years DOI); and a later or “severe and enduring” equivalent stage (commonly defined by 7+ years DOI). There was limited attention to intermediate or middle stages such as partial/full remission stages [45]. Most models described a linear progression across stages. As in Tomba et al., we found little consensus on stage definitions [15] and terminology, particularly for later stages [24, 26, 27, 34, 43, 55].

### Stage modifiers

The current evidence base has not yet explicitly differentiated stage modifiers such as progression factors concerning illness course, or extension factors demonstrating increasing diversity of signs beyond the primary illness [91].

DOI is often used as a proxy for progression, and longer DOI, predominantly within AN samples, was associated with adverse physical outcomes including reduced bone density, fracture risk, cardiovascular strain, gastrointestinal and dental complications, and structural brain changes [25, 33, 35, 53–57, 62, 74]. In other studies, other factors were stronger predictors of treatment outcome than DOI, such as discharge BMI [43], or else DOI was only predictive of outcomes alongside, for example, perfectionistic traits, impairment, or child sexual abuse [32, 39, 41]. Thus, while commonly used, DOI alone does not consistently predict outcome and is an insufficient proxy for stage. As many studies were AN-only, generalizability to other EDs is unclear.

Only a small number of papers empirically tested DOI with other measures in AN or AN-type OSFED presentations [24, 36, 48]. Research on specific cognitive and biological progression factors was limited, with studies tending to identify state–trait distinctions rather than clear, stage-specific biomarkers. The wider literature demonstrates that cognitive impairments may worsen with longer DOI across ED diagnoses [92, 93]. For AN, eye-tracking studies show that attentional avoidance of high-calorie foods increases over time [94], and that structural brain changes are more pronounced and slower to reverse in individuals with longer DOI [95, 96]. These findings are fragmented across individual studies, requiring systematic integration into clinical staging models.

Qualitative studies highlighted dimensions rarely integrated into staging frameworks. Most studies focused on longstanding AN, contributed little to early/recent-onset EDs, and rarely attempted to connect lived experience across illness phases. Identity entanglement, shame, hopelessness, and lost autonomy were frequently cited as characteristic of longstanding AN, as were recurrent treatment failures [64, 66, 70], which may inform progression to this stage.

Across studies, several additional physical and diagnostic features such as cardiovascular strain [54], gastrointestinal and dental problems [74], and metabolic or microbiome disruption [58] represent increased illness complexity beyond core ED symptoms. Under a staging framework, these represent extension factors occurring independently of progression or as a direct consequence of the ED [8]. Wider literature on the longer-term outcomes of EDs also demonstrates physical sequelae such as osteopenia/osteoporosis [97], obesity [1], and adverse reproductive outcomes such as miscarriage, preterm delivery, and pregnancy complications [98].

While some studies demonstrate increased comorbid anxiety [22] and depression [35] over time, some evidence suggested that diagnostic diversity decreases over time, with early-stage presentations shaped by broader psychological vulnerabilities and later stages characterized by more fixed and tightly clustered ED-specific symptoms [40, 51], consistent with qualitative accounts of EDs becoming increasingly entangled with identity over time.

### Treatment implications

Earlier detection and effective intervention appear important for improving outcomes, partly because of the risk of worsening symptoms [23, 25, 33, 34] but also to avoid the need for costly, intensive, and invasive treatments [63, 64, 66, 68, 71]. Some clinical features may help identify those at risk of poorer trajectories at an earlier stage, including purging behaviors and externalizing problems, while delayed intervention has been associated with greater treatment intensity and poorer functional outcomes [22–24]. Notably, shorter-duration AN showed less improvement in body image concerns following inpatient CBT than longer-DOI groups [37], consistent with network analyses suggesting cognitive symptoms are most central in early illness [6], highlighting body image as a key treatment target at this stage.

Longstanding EDs appeared to carry unique treatment needs. Addressing trauma and early adversity was identified as an important treatment target [32, 68, 70, 77], with trauma-informed, autonomy-supportive models specifically recommended [66, 67, 70, 71]. A focus on quality of life and social and occupational functioning were also key treatment targets for this stage, with psychosocial interventions prioritized over weight-focused treatment [64, 74, 75]. Longstanding AN was frequently described as interwoven with identity, whereas a case study exploring identity in the treatment of early-stage AN was found to be effective in reducing the dominance of AN [73]. Further details of the treatment recommendations and clinical implications for each individual study are provided in the Supplementary Tables S4–S6.

### Quality of evidence and suggestions for future research

Most studies were rated as moderate to low strength of evidence. Overall, stronger experimental and multimethod designs were missing. All JBI rankings can be found in Supplementary Tables S4–S6.

Randomized controlled trials (RCTs) evaluating interventions tailored to proposed stages would elucidate whether treatment outcomes differ between stages. Although some trials exist, for example for longstanding AN [99, 100] and for indicated prevention [101], few test interventions across multiple stages of illness, and large-scale, stage-stratified RCTs are scarce [102]. Prospective longitudinal studies using multimodal ([epi]genetic, neural, cognitive and psychosocial) assessments to deeply phenotype participants could inform illness stages and stage transitions, including but not limited to ABCD, IMAGEN, ESTRA, STRATIFY [103–106], and the newly established “STORY” cohort, which also includes novel digital markers [107].

Further research is needed across BN, BED, OSFED, and ARFID, as the evidence focused on AN and few diagnosis-specific conclusions could be drawn. Given high rates of diagnostic crossover in EDs [108], staging frameworks should account for diagnostic changes over time.

### Moving the field forward: A tentative new staging model

**Figure 3.**
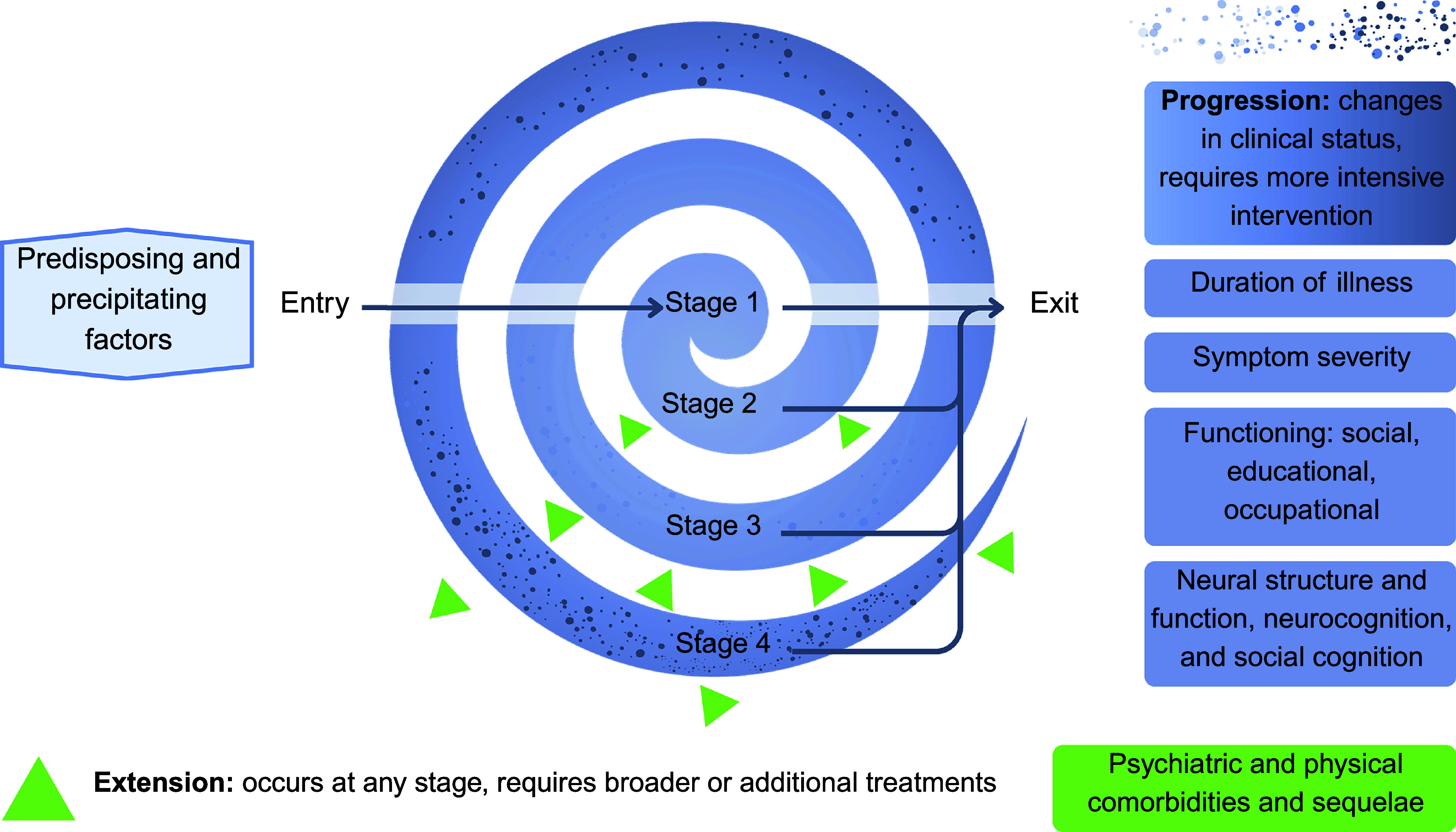
Working staging model for eating disorders. Figure 3. long description.

Based on the findings of this review, we propose a tentative, heuristic staging model for EDs in Figure 3, intended to guide future research rather than serve as a prescriptive clinical tool. The text is inspired by Haas et al. [109] and the model is also informed by parallel qualitative work exploring lived-experience perspectives on staging, which highlights concerns about linear models and rigid “boxes,” supporting a more fluid representation of illness trajectories [110].

A key feature of this staging model is the use of a spiral to reflect the varied trajectories individuals with EDs experience. Four stages were selected, as this is a common configuration in the identified literature and aligns with established staging frameworks in other mental disorders [8, 109]. However, the optimal number of stages is not currently known. Each stage is not a fixed category; transitions reflect changes in clinical status and treatment needs across multidimensional indicators identified in the reviewed evidence, including DOI, symptom severity, psychosocial functioning, cognitive and neurobiological changes, and medical and psychiatric factors. Other configurations may prove more valid as evidence develops. At any point, individuals may require broader or more intensive interventions due to comorbid psychiatric or physical complications. The spiral also includes an all-stage accessible exit point to better showcase that individuals can recover at any stage [111].

Table 6 details some key recommendations to facilitate onward progress in this area.

**Table 6.** Key recommendations for advancing clinical staging in EDs Table 6. long description.

Recommendation	Description
Broaden staging frameworks	Staging frameworks should incorporate biological and psychosocial domains, extending beyond duration and behavioral symptoms.
Advance staging research	Further focused research on staging is needed, including progression and extension factors, with particular attention to middle phases, which may represent critical but under-examined periods and have received less attention than early and later stages.
Evaluate stage-matched interventions	Personalized treatment approaches should be evaluated based on ED stage and associated progression and extension factors.
Promote co-produced, international approaches	International, co-produced approaches with lived-experience expertise are needed to develop, refine, and apply staging frameworks and related concepts in EDs.

Abbreviation: ED, eating disorder.

### Limitations

The included studies used various methods, samples, and terminology, making it difficult to draw firm conclusions about how ED stages should be defined. Conceptual ambiguity and inconsistent language regarding staging or progression meant that staging concepts were often inferred retrospectively, where it was not the intention of the original authors.

Limiting our search to articles published only in the English language raises the possibility of bias. Our study retrieved no studies from Asia or from Africa, and only one study from South America (Chile). In addition, reporting of ethnicity was low across the included studies. Consequently, staging-relevant literature appears to rest on a demographically narrow or, in many cases, ambiguous evidence base. Notably, the Chilean study suggested that DOI criteria may be inappropriate outside Western contexts [29], potentially reflecting differences in healthcare structures, service access, or cultural context, and indicating an absence of contextually informed evidence to guide clinical staging.

Finally, we were unable to complete forward citation searching, meaning some newer studies may have been missed. Given the heterogeneity and conceptual variation in the existing literature, a broader search may have added further complexity to synthesis.

## Conclusion

Clinical staging frameworks can add valuable structure for supporting diagnosis, prognosis, prevention, and tailored intervention in EDs. However, stages are currently inconsistently used, and international collaboration and co-production are required to develop consistent language and criteria [91]. As such, we invite critical appraisal and evaluation of the newly proposed staging model from researchers, clinicians, and those with lived/living ED experience.

## Supporting information

10.1192/j.eurpsy.2026.12241.sm001 Hyam et al. supplementary material Hyam et al. supplementary material

## Data Availability

The data generated from this study are available from Lucy Hyam (lucy.e.hyam@kcl.ac.uk) upon reasonable request.

## Supplementary material

The supplementary material for this article can be found at http://doi.org/10.1192/j.eurpsy.2026.12241.

## Artificial intelligence

AI-assisted tools were employed solely for language editing, including shortening wording. The authors reviewed and approved all content and take full responsibility for the manuscript.

## Long descriptions

### Table 1. Long description

The table consists of two columns: Category and Details.

* Participants: Includes clinical and non-clinical samples with disordered eating or diagnosed E D, including A N, B N, B E D, O S F E D, or A R F I D.

* Concept: Studies comparing defined stages or phases of E D (high-risk, early, or late-stage); proposing a complete staging model; comparing groups based on illness duration; or examining clinical, psychosocial, or biological variables related to illness progression and severity markers.

* Context: Any healthcare or community setting with no geographical restrictions.

* Evidence types: All types of documents and study designs.

* Language: Only English language publications.

Footnote abbreviations: A N (anorexia nervosa), A R F I D (avoidant restrictive food intake disorder), B E D (binge eating disorder), B N (bulimia nervosa), E D (eating disorder), O S F E D (other specified feeding or eating disorder).

Navigate back to Table 1..

### Figure 1. Long description

The flowchart is organized into three vertical phases: Identification, Screening, and Included.

1. Identification Phase:

* Top central box: Records identified (N = 19,682) from PubMed (n = 6,069), O V I D Medline, Psyc I N F O (n = 8,033, plus n = 378 Embase 10 Jan search), and W o S (n = 5,202).

* Right box: Records removed before screening: Duplicate records removed (n = 11,087).

2. Screening Phase:

* Central box 1: Records screened (n = 8,595).

* Right box 1: Records excluded (n = 8,350).

* Central box 2: Reports sought for retrieval (n = 245).

* Right box 2: Reports not retrieved (n = 6).

* Central box 3: Reports assessed for eligibility (n = 239).

* Right box 3: Reports excluded (total n = 179) for the following reasons: No indicator of staging (n = 70), No comparison of illness stages, duration, or staging-relevant outcomes (n = 52), Study design outside of eligibility criteria (n = 24), Non-English language (n = 2), Age of onset comparisons only (n = 7), Focus not staging (n = 23), and Abstract only (n = 1).

3. Included Phase:

* Bottom central box: Studies included in review (n = 60).

Navigate back to Figure 1..

### Table 2. Long description

The table consists of three columns: Category, Subtype, and Count.

1. Quantitative study designs (Total n = 36):

- Clinical trial: 1

- Prospective or retrospective cohort: 2

- Cross-sectional: 15

- Longitudinal: 16

- R C T: 2

2. Conceptual/review designs (Total n = 6):

- Systematic review: 3

- Consensus methods: 1

- Theoretical review: 2

3. Qualitative methods (Total n = 18):

- Meta-synthesis: 3

- Interviews/focus groups: 11

- Photovoice: 1

- Autoethnography: 1

- Case study: 2

4. Qualitative analytic approaches (Total n = 18):

- Narrative approach: 2

- Thematic analysis: 9

- Grounded theory: 1

- Phenomenological analysis: 3

- Descriptive analysis: 1

- Meta-ethnography: 2

Note: R C T stands for randomized controlled trial.

Navigate back to Table 2..

### Figure 3. Long description

The central feature is a purple, textured spiral with four numbered stages. At the core is Stage 1. Moving outward through the spiral layers are Stage 2, Stage 3, and finally Stage 4 at the outermost perimeter.

Horizontal flow:

- On the far left, a hexagonal box labeled Predisposing and precipitating factors leads to an Entry arrow.

- The Entry arrow passes through the spiral layers directly to Stage 1.

- From each stage (1 through 4), horizontal lines emerge to the right, curving upward to converge at a single Exit point on the right side of the spiral.

External elements:

- Green triangles point inward toward the spiral at various points. A legend at the bottom defines these as Extension: occurs at any stage, requires broader or additional treatments.

- A green box at the bottom right is labeled Psychiatric and physical comorbidities and sequelae.

Vertical list on the right:

- A column of blue boxes defines Progression as changes in clinical status, requires more intensive intervention.

- Below this, criteria for progression include: Duration of illness, Symptom severity, Functioning (social, educational, occupational), and Neural structure and function, neurocognition, and social cognition.

Navigate back to Figure 3..

### Table 3. Long description

The table is organized into three columns: Category, Staging criteria, and Details (number of groups, stage label).

1. Category: 1. Clinical Scores. Staging criteria: Symptom or diagnostic status. Details include subthreshold versus full E D using symptomatic assessments (Child E D E-Q and E D E-Q), prodromal versus full E D onset using diagnostic interviews (E D D I), and recovery, remission, or relapse criteria.

2. Category: 2. D O I-only stages. Staging criteria: D O I grouping only. Details are split into two groups and three groups. Two-group examples include less than 3 years (short duration) versus greater than 3 years (long duration), and less than 7 years (non-S E-A N) versus greater than 7 years (S E-A N). Three-group examples include onset, established, and recovered; or less than 3 years (short), 3 to less than 7 years (medium), and greater than 7 years (long).

3. Category: D O I plus another variable. Staging criteria: D O I combined with other clinical variables. Details include two groups (e.g., D O I less than 3 years plus D A S S score), three groups (e.g., E D E-Q severity plus D O I), and four groups operationalizing multidimensional severity into Stage 1 (Mild A N, less than 6 months), Stage 2 (Moderate A N, 6 months to 2 years), Stage 3 (Severe A N, 2 to 5 years), and Stage 4 (Extreme A N, greater than 5 years).

4. Category: Single stage plus predictors. Staging criteria: One stage assessed with other indicators. Details include S E-A N classification tested via severity and chronicity indicators.

Navigate back to Table 3..

### Table 4. Long description

The table consists of two columns: Category and Summary.

Under the Category ‘D O I associations with outcomes’, the Summary column lists the following points:

* Analyzed across pre-defined outcome groups or examined as a continuous variable with outcomes.

* Compared across three outcome groups defined by dietary restriction changes.

* Compared across four outcome groups defined by B M I trajectory.

* Four outcome groups in longstanding E D based on prior treatment response; associated with four outcome groups, with personality and psychopathology; associated with two outcome groups.

* Associated with E D severity and treatment-seeking behavior.

* Associated with outcomes and explored for potential staging.

* Associated with myocardial mechanics; symptomatic assessments used to identify relapse predictors.

* D O I and amenorrhea duration associated with bone mineral density and hip strength.

* Associated with bone mineral density.

* Associated with insular cortex volume; personality assessed across four long-term outcome groups defined by clinical status.

Abbreviations provided at the bottom: B M I stands for body mass index; D O I stands for duration of illness; E D stands for eating disorder.

Navigate back to Table 4..

### Table 5. Long description

The table is organized into three columns: Category, Staging criteria, and Details (number of groups, stage label).

1. Category: Clinical Scores. Staging criteria: Symptom, diagnostic, or polygenic status. Details: Subclinical to full-threshold B N using symptomatic assessments E D I-3 and S C I D-I V. High versus low polygenic risk score for A N and differences in illness trajectories.

2. Category: D O I (Duration of Illness). Staging criteria: D O I grouping only. Details: Lists various timeframes including less than or equal to 3 years (early-stage A N), less than 3 years (early-stage E Ds), greater than 3 years (longstanding A N or E Ds), greater than or equal to 5 years (longstanding B N), greater than or equal to 7 years (longstanding A N), greater than or equal to 10 years (longstanding A N), 14 to 28 years (longstanding A N), 18 years (longstanding A N single case), greater than 20 years (longstanding A N), 25 years (longstanding A N single case), and an average of 6.3 to 22.4 years for all E Ds.

3. Category: D O I plus other criteria. Details: One group defined as greater than or equal to 7 years plus at least 1 inpatient treatment plus severe impairment across two life domains.

4. Category: Named stage. Details: One group where criteria are not specified for all E Ds.

Navigate back to Table 5..

### Table 6. Long description

The table consists of two columns titled Recommendation and Description.

* Row 1: Broaden staging frameworks. Description: Staging frameworks should incorporate biological and psychosocial domains, extending beyond duration and behavioral symptoms.

* Row 2: Advance staging research. Description: Further focused research on staging is needed, including progression and extension factors, with particular attention to middle phases, which may represent critical but under-examined periods and have received less attention than early and later stages.

* Row 3: Evaluate stage-matched interventions. Description: Personalized treatment approaches should be evaluated based on E D stage and associated progression and extension factors.

* Row 4: Promote co-produced, international approaches. Description: International, co-produced approaches with lived-experience expertise are needed to develop, refine, and apply staging frameworks and related concepts in E D s.

A footer notes that E D stands for eating disorder.

Navigate back to Table 6..
