# Supplementary material for: Clinical staging for eating disorders: A scoping review and update
Source: Eur Psychiatry. 2026 Jul 21;69(1):e77. doi: 10.1192/j.eurpsy.2026.12241 (PMC13458303; doi:10.1192/j.eurpsy.2026.12241)
Supplement: Supplementary file 1 [file S092493382612241Xsup001.docx]

**Supporting information**

**Supplementary Table 1.** Updated data extraction table with minor modifications from the protocol.

| **Data item** | **Description of data item** |
| --- | --- |
| Author(s) | Names of the authors of the document. |
| Year of publication | Year document was published. |
| Type of document | Description of type of document, for example primary research, review, protocol paper, theoretical paper, opinion piece, editorial. |
| Title | Title of document. |
| Location | Where the document/study was published. |
| Aim(s)/research question/purpose | Summary of the document’s purpose or research aim(s). |
| Study design | Design of study including randomised controlled trial, cohort studies (prospective and retrospective), cross-sectional studies, case studies/series, etc. |
| Population of interest, sample size (if applicable), key demographics | If a primary/secondary research study, details of the sample studied, including sample size, reported age, and other demographic information. |
| Methodology | E.g., qualitative, quantitative, or mixed methods. |
| Setting/context of study | If applicable, the setting of the study where research took place. For example, if data were collected in schools or in hospitals. |
| Eating disorder diagnostic threshold | Including high risk/disordered eating behaviour, or full eating disorder. |
| Eating disorder diagnosis ascertainment | Diagnostic or symptom criteria used. |
| Clinical staging attempt | E.g., full model, partial model, single stage. |
| Main results/findings | Can include outcome measures related to illness stage such as quality of life or biological findings. |
| Stage(s) of illness defined or examined | E.g., high-risk, prodromal, early-stage illness, full syndrome, long-lasting illness. Or, can include ~~age groups or~~ duration of illness assessment. |
| Potential intervention/ implications for treatment (if applicable) | E.g., stage-tailored treatment adaptations, specially designed services (e.g., early intervention services) or treatment/prevention types. |

**Note**. Strikethrough text indicates changes to published protocol.

**Supplementary table 2. Examples of excluded study designs.**

**Exclusions**

Comparisons between weight-restored and underweight patients or pre- and post- weight change in the same sample (without descriptors of stage or illness duration).

Secondary research commentaries or book chapters on treatment pathways or specific stages with no new data/models presented

Qualitative studies not specific to staging, for example, experiences of family-based therapy, or caregiving experiences not specific to stages.

**Supplementary Table 3. Search terms**

(("Anorexia Nervosa"[MeSH Terms] OR "Anorexia"[MeSH Terms] OR "Avoidant Restrictive Food Intake Disorder"[MeSH Terms] OR "Binge-Eating Disorder"[MeSH Terms] OR "Bulimia Nervosa"[MeSH Terms] OR "Bulimia"[MeSH Terms] OR "Feeding and Eating Disorders"[MeSH Terms]) OR (eating disorder*[tiab] OR binge-type[tiab] OR anorexi*[tiab] OR bulimi*[tiab] OR binge-eating*[tiab] OR binge eating*[tiab] OR eating disorder not otherwise specified[tiab] OR EDNOS[tiab] OR disordered eating[tiab] OR other specified feeding[tiab] OR OSFED[tiab] OR avoidant restrictive food intake disorder [tiab] OR ARFID [tiab] OR unspecified eating disorder [tiab]))

AND (clinical stag*[tiab] OR stag* model[tiab] OR stag* paradigm[tiab] OR trajector*[tiab] OR *progression[tiab] OR at-risk[tiab] OR early stage[tiab] OR early onset[tiab] OR prodromal[tiab] OR illness stage[tiab] OR late stage[tiab] OR “severe and enduring”[tiab] OR persistent[tiab] OR longstanding[tiab] OR long-standing[tiab])

NOT (cancer[ti])

### **Supplementary information 1. Modifications to the protocol**

We decided to include manuscripts only if they clearly characterised stages or were well-defined enough to inform clinical staging. Given our main interest in differences between stages, a new focus on comparative studies was defined. Manuscripts that compared groups based on defined stages, by illness duration, or by diagnostic status (e.g., ‘subthreshold’ vs diagnosed) were included. Therefore, studies on risk factors or at-risk populations were only included if compared to a later diagnostic stage.

In addition, studies testing treatments for certain stages were only included if they also compared outcomes to another stage. Studies on single stages without another stage as a comparison group were excluded. Criteria were also changed so that studies comparing groups by age/age of onset were not included unless they specifically used this criterion within the context of illness stages, due to a high volume of studies retrieved that compared groups by age/age of onset. We decided not to impose these restrictions for qualitative research, including studies that focused on illness journeys or experiences within one stage only (e.g., experiences of recent onset illness) in an attempt to prioritise lived experience perspectives. We also excluded reviews on single stages or treatment pathways that provided no new data (e.g., reviews on long-standing EDs), or that did not propose a new staging or progression model within the review. Documents on the role of genetics were also excluded from the main analysis unless they could be directly related to staging. Examples of study designs and research concepts for exclusion are given in Appendix G (Supplementary methods).

Two final modifications are that first, we included studies up to January 2026 instead of the date identified in the protocol, to incorporate as much emerging evidence as possible. We were not able to invite youth advisors from the EDIFY consortium to provide commentary and feedback on the review.

**Supplementary Table 4. Quantitative papers data extraction**

| **Author(s), Year, Location** | **Study design** | **Population** | **Diagnosis** | **Clinical staging** | **Key findings** | **Potential intervention/ implications for treatment** | **JBI Levels of Evidence** |
| --- | --- | --- | --- | --- | --- | --- | --- |
| Abber et al. 2025 USA | Longitudinal | N = 276, 91% female, 84% white x̄ age = 18.0 years (SD = 6), DOI range = 3.7 years - 6.5 years | ARFID, AN | DOI (no groups) across outcome groups. | Longer DOI and higher anxiety differentiated those with a less favourable outcome (good response, rebounding) from those with a more favourable outcome (good response or gradual response, low symptoms). | Clinical features at baseline could help to identify those at risk of worse outcome, allowing for intervention planning at discharge. | Prognosis - 3b |
| Allen et al. 2013 Australia | Longitudinal | N = 1,383, 51% female Followed from ages 14 through 20 | Full, partial, and subthreshold AN, BN, BED and PD | Subthreshold - full ED using symptomatic scores. | Of individuals with ED at age 14, 44% remitted and did not relapse by age 20, 37% experienced a later or partial remitting/relapsing course, and 19% had persistent EDs at all three time points. Purging at 17 and externalising behaviour problems at age 14 were the strongest predictors of ED persistence at age 20. Participants who did not complete high school more likely to have persistent ED and those with persistent ED had more pronounced depressive and anxiety symptoms at age 20. | Early detection and intervention recommended for adolescents as symptom remission reduced after age 17 and persistent ED pathology increases risk for poor functioning at age 20. Purging, no high school completion, and externalising behaviour problems may be characteristic of ED persistence. | Prognosis - 3b |
| Ambwani et al. 2020 UK | Longitudinal | N = 187, 96.8% female, 97.5% white x̄ age = 27.81 (SD = 9.30) years | AN or OSFED with AN features | DOI groups and emotional distress: Early stage AN (< 3 years DOI) and severe and enduring AN (>7 years DOI & DASS score ≥60). | At 12 months, severe and enduring AN group were more likely to receive intensive care, had worse ED symptoms, and worse work and social wellbeing compared to early-stage AN. SE-AN group also had less improvement in work and social wellbeing over time. | Differences in course and service use between different groups suggest a need to consider staging in clinical decision-making: DOI & distress may be considered as indicator of illness severity and poorer psychological wellbeing in longer DOI may need specific treatment efforts. | Prognosis - 3b |
| Andrade et al. 2017 Portugal | Retrospective cohort study | N = 169, 94.1% female, x̄ age = 22.61 (SD = 7.95) years; x̄ DOI AN binge/purge = 6.05 (SD = 6.5) years, AN restricting = 3.13 (SD = 3.97) years | AN | DOI groups: <1 year, 1-2 years, >2 years. | In <1 year DOI group, 53.8% had a poor outcome, in the 1-2 years DOI group, 68% had a poor outcome, and in the > 2 years DOI group, 77.3% had a poor outcome. Only 14.8% of patients achieved full remission. Longer DOI significantly predicted a worse outcome, however, there were no significant differences in dropout rates between DOI groups. | Significant differences in patients’ outcome according to DOI suggest early intervention is critical. There is also a need for better strategies for longer DOI AN patients, and no support to suggest worse treatment engagement in this group. | Prognosis - 3b |
| Bass et al. 2005 Australia | Retrospective and prospective cohort study | N = 31, 100% female, 100% white, age range 13.4 - 18 years at onset. x̄ DOI 'established AN' for 'disease' cohort = 2.5 years , recovered cohort = 1.7 years | AN | DOI groups: onset, established cohort mean duration 2.5 years; recovery cohort established AN mean duration 1.7 years. | Bone density loss increased with DOI (−0.6 SD after 0.8 years; −1.0 SD after 2.5 years), but improved with recovery; in the recovered cohort, bone density declined by −1.9 SD after 1.7 years of illness, but showed a +1.5 SD improvement after 2.7 years of recovery. | Increasing DOI causes decline in bone health but recovery of body weight and return of regular menses can restore normalisation of bone density changes. Early intervention could prevent worsening physical complications. | Prognosis - 3b |
| Calugi et al. 2013 Italy | Clinical trial | N = 95, 100% female x̄ age = 25.7 years (SD = 9.1), median DOI = 6 years (IQR = 13.3 years; range = 0.5–36 years) | AN, EDNOS | DOI groups: shorter ED (<10 years, longstanding ED (≥10 years). | At admission, longstanding ED patients had significantly higher age, BMI, and purging behaviours compared with shorter ED patients, and no differences in ED or general psychopathology, or personality traits. There were no differences between DOI groups in treatment dropout or inpatient CBT outcomes (BMI, ED and general psychopathology). | Longer DOI patients have different ED presentation compared with shorter DOI patients (e.g., more purging behaviours). However, outcome with intensive inpatient program of CBT is not different between groups. | Effectiveness - 3c |
| Calugi et al. 2017 Italy | Longitudinal | N = 66, 97% female x̄ age = 26.1 (SD = 5.9) years, x̄ DOI = 7.7 years (SD = 5.7) | AN | DOI groups: non-severe enduring AN (< 7 years), severe and enduring AN (> 7 years DOI). | Both DOI groups improved in BMI and ED and general psychopathology during treatment. At 12 month follow up, both groups showed similar rates of good BMI outcome and minimal ED psychopathology. No differences in treatment dropout between groups. | Recovery-based treatment such as inpatient CBT-E effective for patients with both SE-AN, and non-SE-AN. | Effectiveness - 3c |
| Dancyger et al. 1997 USA | Longitudinal | N = 52, 100% female  x̄ age = 19.29 years (SD = 4.97) | AN | Personality differences among longitudinal outcome groups. | Significant improvements in MMPI scores from admission to discharge and follow-up. Outcome category significantly associated with MMPI change over time; recovered individuals had greater reductions than those with poor outcome. Clinical elevations at follow-up were most common in poor outcome group. | Persistent personality features are best measured following acute AN treatment. | Prognosis - 3b |
| Dapelo et al. 2020 Chile | Cross-sectional | N = 41, 100% female x̄ age = 28.93 years (SD = 8.31, range 18 - 51) x̄ DOI = 119.27 months (SD = 93.15, range = 12–317) | AN, BN, BED & OSFED | DOI (no groups). | No significant association between DOI and severity but DOI significantly associated with delays seeking treatment. For each month of delay, DOI increased by 0.87 months. | Late stage illness cannot be defined by DOI alone, other criteria (time to access treatment) should also be considered. | Effectiveness - 4b |
| Dare et al. 1990 UK | Randomised controlled trial | N = 80, 90.4% female x̄ age = 21.8 years (SD = 7.1) , x̄ DOI = 3.8 years (SD = 3.1) | AN, BN | DOI and age of onset groups: (1) Age of onset <18 years and DOI <3 years, (2) Age of onset <18 years and DOI >3 years, (3) Onset >18, (4) patients with BN. | Outcome with family therapy better for early onset, short DOI group. For shorter onset, longer DOI group, no differences between family and individual therapy outcome. | Staging may guide treatment selection in that family therapy is more effective for younger patients, particularly with early onset and short DOI. | Effectiveness - 1c |
| Di Lodovico et al. 2024 France | Longitudinal | N = 181, 94.5% female x̄ age = 27.5 years (9.50) x̄ DOI = 9.66 years (SD = 9.56) | AN | DOI (no groups) across outcome groups. | Four BMI trajectory classes identified, suggesting different stages of illness severity, chronicity, and treatment response in AN. Class 1 with higher baseline BMI showed slow, plateauing weight gain and a high prevalence of binge-purge AN and complex behavioural symptoms. Class 2 included patients with longer DOI, longer inpatient stay, and higher nasogastric feeding support, who appeared more chronic with complications, but terms such as “refractory” or “enduring” not appropriate as they ultimately achieved comparable discharge weight gain. Class 3 demonstrated a rapid early weight gain and higher anxiety symptoms. Class 4, with younger patients and fewer previous hospitalisations, had the lowest admission weight and the most rapid and sustained weight gain, indicating a more responsive group. | BMI trajectory classes demonstrate variations in illness severity, chronicity, and treatment response among AN patients. Individual differences such as comorbidities influence outcomes. 'Enduring-ness' term may not be appropriate for longer DOI stage, due to comparable weight gain and remission to other classes. | Effectiveness - 3c |
| Eielsen et al. 2023 Norway | Longitudinal | N = 84,  x̄ age = 29.9 years (SD = 7.5), x̄ DOI = 13.3 years (SD = 8.0) | AN, BN, & OSFED | Longitudinal outcome groups in one stage: Longstanding ED (patients receiving prior treatment without improvement, average DOI 13.3 years). | Four different symptom trajectories identified. Childhood sexual abuse was overrepresented in patients with persistently high ED symptoms and also significantly lowered probability of a favourable long-term ED outcome. | Longstanding EDs have diverse trajectories. Trauma informed care particularly important for longstanding EDs. | Prognosis - 3b |
| Erdur et al. 2017 Germany | Cross-sectional | N = 64, 100% female x̄ age acute AN group = 22.13 years (SD = 3.8), chronic AN group = 34.7 years (SD = 9.3), recovered AN = 40.8 years (SD = 6.8) and healthy controls = 29 years (SD = 8.3) | AN | DOI groups: Acute AN (<5 years), chronic AN (≥5 years), recovered AN (>5 years recovered) | Healthy controls showed startle response matching subjective rating, however, participants with acute and chronic AN showed mismatch between startle response and subjective ratings. This mismatch was significant only when comparing chronic AN to healthy controls. Recovered AN participants also showed no modulation of startle matching their ratings, suggesting normalisation with recovery. | This emotional processing alteration is a state-dependent feature of AN resolving with recovery, little support for stage-specific differences. | Effectiveness - 4b |
| Favaro et al. 2015 Italy | Longitudinal | N = 96, 100% female x̄ age AN group = 26.1 years (SD = 7.2), recovered group = 26.3 years (SD = 7.1), x̄ DOI AN group = 78.6 months (SD = 81.3), recovered group = 45.7 months (SD = 65.0) | AN | DOI (no groups) across outcome groups. | Patients with a poor outcome at 3 years had significantly lower baseline gyrification compared to healthy controls and recovered AN patients at follow-up after controlling for DOI and grey matter volume. Gyrification not correlated with BMI, amount of weight loss, onset age, or DOI for AN patients. | Abnormal brain gyrification may be a predictor of poor AN outcome, but these alterations cannot be considered an endophenotype/biomarker for identification of the disease (state-related, little support for stage-specific differences.) | Prognosis - 3b |
| Fernández‐Aranda et al. 2021 Spain | Longitudinal | N = 1199, 90.1% female x̄ age = 27.6 years (SD = 9.0), x̄ DOI = 7.7 SD = 7.3 years | AN, BN, BED, & OSFED | DOI (no groups) across outcome groups. | Outcome for all EDs except BED associated with DOI and number of previous treatment attempts. The DOI associated with at least 50% likelihood of poor outcome was 6 years for OSFED, 12 years for AN, 13 years for BN, and 21 years for BED. However, no clear criteria for staging using DOI emerged. Higher perfectionism, higher likelihood of tobacco and alcohol misuse also associated with poorer therapy response. | Optimising treatment response in early illness may prevent chronicity, particularly for those with so-called 'milder' ED symptoms (OSFED). | Prognosis - 3b |
| Herzog et al. 2022 Germany | Randomised controlled trial follow-up | N = 242, 100% female, 100% white x̄ age = 32.4 years at follow up | AN or subsyndromal AN | DOI groups ≤6 vs >6 years at baseline. | Significant baseline predictors of BMI at the 5-year follow-up were higher BMI, shorter DOI (≤6 years), and no depression diagnosis. | Treatment should target earlier stage of illness and also need to consider other comorbidities such as depression. | Effectiveness - 1c |
| Kiely et al. 2026 | Cross-sectional | N = 208, 93% female, 84% white, median age = 24.0 (IQR 19–32) | AN | DOI groups: Early stage AN (≤3 years DOI) and longstanding AN (≥7 years DOI). | No group differences in illness severity, but longer duration group had more comorbidities, longer treatment delays, more treatment episodes, and poorer treatment experiences. | Lack of treatments may have an inadvertent impact on DOI, evidence-based treatments tailored to needs and preferences are required. Repeated unhelpful treatments may undermine hope and protract illness. | Effectiveness - 4b |
| Krantz et al. 2024 USA | Cross-sectional | N = 29, 97% female, 90% Caucasian x̄ age = 27.7 years (SD = 7.6), x̄ DOI = 9 years (range = 5-18 years). | AN | DOI (no groups) and outcome. | Longer DOI was significantly associated with worsening of all global and regional measures of myocardial deformation. | Incorporating strain imaging into routine health checks for those with severe AN with longer DOI may help identify those at greater clinical risk. | Effectiveness - 4b |
| Lin et al. 2021 USA | Cross-sectional | N =145, 87% female, 89% white x̄ age = 16.4 years (SD = 3.0), DOI 19% ≤ 6 months, 15% 7–12 months, 65% >1 year | AN, atypical AN, OSFED, ARFID | DOI groups (≤6 months, 7-12 months, >1 year) as predictors of psychiatric comorbidities. | Longer DOI predicted higher depression scores & higher pre-morbid BMI (≥75th percentile) was linked to lower depression scores but not anxiety scores. Malnutrition was not an independent predictor of anxiety or depression. | Early identification and multidisciplinary intervention beyond weight restoration may be necessary, particularly for those with prolonged illness or lower pre-morbid BMI. | Effectiveness - 4b |
| Lopes et al. 2024 UK | Cross-sectional | N = 94, 100% female x̄ age = first-episode group 24.2 years (SD = 5.9), persistent AN group 29 years (SD = 8.6), x̄ DOI = first-episode group 1.9 years (SD = 0.8), persistent AN group 15.3 years (SD = 8.5). | AN | DOI groups: First episode AN (≤ 3 years DOI) and persistent AN (≥ 7 years DOI). | Women with both first episode and persistent AN were more likely to report a fracture compared to HC (33.3% and 37.8% respectively), irrespective of age. | Increased fracture risk present even in early stages of AN. | Effectiveness - 4b |
| Maguire et al. 2017 Australia | Longitudinal | N = 171, 98% female x̄ age = 24.4 years (SD = 8.1), x̄ BMI = 16.5 (SD = 2.32), x̄ DOI = 7.97 years (SD = 7.55) | AN, EDNOS-AN | CASIAN four stage model of severity scores: Stage 1 (subclinical symptoms), Stage 2 (mild AN, outpatient treatment), Stage 3 (severe AN, hospitalization), Stage 4 (extreme AN, long-term care). | CASIAN significantly distinguished milder (Stages 1–2) from more severe AN (Stages 3–4). Stage was significantly associated with treatment intensity (more likely to be in hospital treatment, less likely to be in outpatient care) and DSM-IV-R diagnosis (Stage 1 and 2 more likely EDNOS, those in Stage 3 and 4 illness more likely to be full-criteria diagnosis [although DSM 5 reclassification captured all as full-syndrome AN]). | Staging may help guide treatment intensity and prognosis for AN patients. | Prognosis - 3b |
| Marzola et al. 2021 Italy | Cross-sectional | N = 169, 93% female, 100% Caucasian x̄ age short DOI group 19.6 years (SD = 4.9), medium DOI group 23.1 years (SD = 7.5), long DOI group 31.7 years (SD = 9.4) | AN | DOI groups: <3 years: short duration AN, 3-<7 years: medium duration AN, ≥7 years: long duration AN | DOI did not predict severity, but longer DOI was linked to more hospitalisations and prior treatment failures. All groups improved post-hospitalisation, but short-duration AN improved less in body image concerns. | DOI alone may not define SE-AN; treatment approaches should consider multiple factors beyond DOI. Shorter DOI AN may need targeted body image interventions. | Effectiveness - 4b |
| Meneguzzo & Todisco 2024 Italy | Longitudinal | N = 360, 100% female x̄ age = 26.10 years (SD = 10.36), x̄ DOI acute ED group 1.83 years (SD = 0.93), SE-ED group = 16.21 years (SD = 8.08) | AN, BN, or OSFED | DOI groups: Acute ED (< 3 years DOI), SE-ED (>7 years DOI). | Treatment completion and response similar between DOI groups. Compulsive self-monitoring higher in the acute ED than SE-ED group at baseline, but SE-ED group showed a greater reduction in compulsive self-monitoring over treatment. | Role of compulsive self-monitoring (the compulsive checking of physical appearance) differs according to DOI, reflecting a habit-based or anxiety-related behaviour supporting treatment engagement in longer DOI. | Effectiveness - 3c |
| Nilsson et al. 2008 Sweden | Longitudinal | N = 68, 100% female Median age initial assessment = 15 years (min 10–max 17), 1st follow up = 23 years (min 18-max 25), 2nd follow-up = 30 years (min 24–max 36), median DOI before assessment = 6 months (min 1–max 36 months) | AN | Longitudinal outcome groups. | Perfectionism remained stable over time, even as ED and psychiatric symptoms decreased during recovery. Higher initial perfectionism was associated with longer DOI. | Clinicians should monitor perfectionism in AN patients as a potential predictor of prolonged illness and relapse risk. | Prognosis - 3b |
| Perkins et al. 2007 UK | Cross-sectional | N = 146, 98.6% female x̄ age shorter DOI sample =17.6 years (SD = 1.7), x̄ age longer DOI sample = 30.4 years (SD = 12.5) | BN and EDNOS-BN | DOI groups: <3 years: short duration vs. ≥3 years: long duration. | No significant differences between shorter and longer DOI BN in terms of stage of change, motivation, confidence and readiness to change. However, relationship between motivational measures, illness severity, duration and comorbidity differed between shorter and longer DOI groups. For longer DOI group, anxiety and depression were negatively associated with DOI (longer DOI = less comorbid symptoms reported). | Readiness to change is not necessarily lower in early illness but longer DOI may be linked to higher anxiety, depression, and symptom severity. However, less comorbid symptoms reporting over time in longer DOI group suggests that these individuals adapted to illness. | Effectiveness - 4b |
| Poet et al. 1993 France | Cross-sectional | N = 18, 100% female, 100% white  x̄ age = 20 years (SD = 6), x̄ DOI = 45 months (SD = 53) | AN | DOI and duration of amenorrhea (no defined groups) and outcome. | AN patients had significantly lower BMD than controls; longer amenorrhea duration correlated with more severe bone loss but not AN duration, suggesting oestrogen deficiency is key in AN-related osteoporosis. | Osteoporosis risk should be closely monitored in AN patients with amenorrhea; hormonal deficiency appears to be a primary factor in bone loss. | Effectiveness - 4b |
| Prochazkova et al. 2025  Czech Republic | Cross-sectional | N = 62, 100% female, Median age acute AN = 29 years, (IQR = 18, 26), severe and enduring = 27 years (IQR = 21.5, 31), median DOI acute AN = 24 months (IQR = 15, 35), SEAN = 102 months (IQR = 84, 150); x̄ BMI acute AN = 15.60 (SD= 1.33), SEAN = 14.14 (SD = 2.31) | AN | DOI groups: acute AN DOI <3 years, severe and enduring AN DOI >7 years. | Greater antidepressant use and somatic anxiety in severe and enduring AN as well as greater microbiota instability and intestinal mucosal disruption. Mass‑spectrometry metabolomics reveal reduced microbial metabolites in both groups. | Microbiome and metabolic profiles differ across stages, and may help indicate progression from acute to chronic stages of AN. | Effectiveness -4b |
| Ramos et al. 2022 Portugal | Cross-sectional | N = 139, 92% female x̄ age = 23.8 years (SD = 8.7), x̄ BMI = 16.07 (SD = 2.3), x̄ DOI = 62 months (SD = 74.8) | AN | DOI groups: (<7 years: non-severe and enduring AN vs. ≥7 years: severe and enduring AN); alternative classification also considered clinical impairment (CIA total score ≥16). | No significant differences in eating pathology or distress based solely on DOI; however, SE-AN group with CIA score ≥16 showed higher psychopathology. | Combining DOI with impairment measures may improve SE-AN classification; illness duration alone may not be a useful staging criterion. | Effectiveness - 4b |
| Raykos et al. 2018 Australia | Longitudinal | N = 134, 97% female Median age = 22 years (IQR = 18, 25), median DOI 5 years (IQR = 3, 12), x̄ BMI 16.8 (SD = 1.5) | AN | DOI groups: 3, 7, or 11 years groups. | Illness severity and duration did not predict treatment outcomes in CBT-E. Lower pre-treatment BMI was associated with greater BMI gains. | Clinicians should offer CBT-E to all AN patients regardless of DOI; the concept of SE-AN may lack clinical utility based on these data. | Effectiveness - 3c |
| Redgrave et al. 2021 USA | Longitudinal | N = 191, 100% female, 88.7% Caucasian x̄ age = 32.5 years (SD = 12.29), x̄ DOI = 13.2 years (SD = 11.6) | AN, subthreshold and OSFED | DOI groups: short-term (<7 years) vs. long-term ill (≥7 years) groups. | Discharge BMI, not DOI, was the strongest predictor of BMI at 6-month follow-up; long-term ill patients responded similarly to short-term ill. | Targeting discharge BMI ≥19 kg/m2 may improve long-term outcomes for AN inpatients; chronicity alone should not define SE-AN. | Prognosis - 3b |
| Richard et al. 2005 Germany | Longitudinal | N = 1,171, AN sample 96.7% female, BN sample 97.7% female x̄ age AN sample = 24.8 years (SD = 5.6) years, x̄ age BN sample = 25.9 (SD = 6.3) years. x̄ DOI AN sample = 5.7 years (SD = 5.3), x̄ DOI BN sample = 8.2 years (SD = 6.1). | AN and BN | Recovery, remission (symptom-free status for a period of time) and relapse (return to full symptomatic status after partial/full remission). | 32.6% of AN and 37.4% of BN patients relapsed within 2.5 years; highest risk of relapse occurred in first 6-7 months post-remission. Low desired weight, long DOI and low severity of ED symptoms associated with higher risk of relapse in AN but not in BN. | Relapse prevention should focus on the first 6-7 months post-remission; predictors like desired weight and EDI scores could guide interventions. | Prognosis - 3b |
| Schorr et al. 2019 USA | Cross-sectional | N = 103, 100% male, 94-100% white  x̄ age AN sample = 30.7 years (SD = 14.2), x̄ age ARFID sample = 27.5 years (SD = 12.4), x̄ age Atypical AN sample = 26.2 years (SD = 8.3), x̄ DOI AN sample = 9.8 years (SD = 10.7), x̄ DOI ARFID sample = 3.4 years (SD = 3.1), x̄ DOI Atypical AN sample = 5.3 years (SD = 5.5) | AN, ARFID, and subthreshold | DOI (no groups) and outcome. | Men with AN had significantly lower BMD than controls; lower muscle mass, longer DOI, and vitamin D deficiency were associated with worse bone health. | Bone health assessments should be prioritized in men with EDs, especially those with prolonged illness duration or low muscle mass. | Effectiveness - 4b |
| Stice et al. 2021 USA | Longitudinal | N = 1,952, 100% women, 66% white x̄ age=19.7 years (SD = 5.7) | AN, BN, BED, and PD | Prodromal - full ED using symptomatic scores. | Compensatory behaviours typically emerged first in AN, BN, and PD, whereas binge eating emerged first in BED; prodromal symptoms strongly predicted disorder onset. | Prevention programmes targeting prodromal symptoms may reduce ED onset. | Prognosis - 3b |
| Tomei & Tomba 2026  Italy | Cross-sectional | N = 139, 85.9% female at‑risk group, 89% female acute group, 100% female clinical group  x̄ age = at-risk group = 27 years (SD = 8.63), acute group = 30.4 years (SD = 9.8) (acute), clinical group = 24.2 years (SD = 10.4)  x̄ BMI = at risk group 23.4 (SD = 4.6), acute group = 22.8 (SD = 4.8), clinical group = 22.0 (SD = 5.8) | AN, BN, BED, OSFED | DOI groups or EDE‑Q severity:<2.3 HC; 2.3–3.4 at‑risk group; ≥3.5 acute group; clinical group: DOI <7 years. | Symptom networks grew denser from at‑risk to clinical stages, shifting from broad transdiagnostic vulnerabilities (stress, depression, irrational beliefs) to tightly clustered ED‑specific symptoms (restriction, weight/shape concerns). Network tests indicated structural and strength differences reflecting increasing rigidity and self‑reinforcement. | Supports stage‑specific treatment planning; early stages require targeting stress, mood symptoms, and cognitive rigidity to prevent network consolidation; later stages may require interventions that disrupt rigid ED‑specific symptom loops. | Effectiveness – 4b |
| Wildes et al. 2017 USA | Cross-sectional | N= 355, 95% female, 94.1% white x̄ age = 26.0 years (SD= 9.2), x̄ DOI = 8.8 years (SD = 8.4) | AN | Tested SE-AN classification via associations among severity and chronicity indicators. | No evidence for a chronic subgroup of AN - indices of chronicity varied dimensionally within each class. Hybrid, three-factor (dimensional), two-profile (categorical) mixture model provided best fit. Factor 1 = ED behaviours; Factor 2 = quality of life domains; Factor 3 = DOI, number of hospitalizations, and admission BMI. Profiles differed on ED behaviours and quality of life but not on indicators of chronicity or BMI. Factor scores predicted outcome at discharge from treatment but not profile membership | SE-AN is dimensional and multifaceted, and is best conceptualised as a hybrid of two severity groups (higher vs. lower) and three continuous dimensions: ED behaviours, quality of life, and chronicity (DOI, hospitalisations, BMI). | Effectiveness - 4b |
| Zucker et al. 2017 USA | Cross-sectional | N = 21, 100% female, 82.6% white (healthy controls n = 20) x̄ age AN sample = 17.48 years (SD = 1.72), x̄ DOI = 3.14 years (SD = 2.03) | AN | DOI (no defined groups). | Right anterior insula volume decreases with longer DOI. Right posterior insula volume increases with longer DOI. | Altered insular cortex structure may contribute to body image disturbance in AN. Larger right posterior insular volume may be explored as a putative marker of illness progression or as a vulnerability factor for illness severity. Future research should explore targeted neurobiological interventions. | Effectiveness - 4b |

*Notes*. The terms used to describe phases or stages of EDs are mirrored to how those authors described their sample.

ED = eating disorder, AN = anorexia nervosa, ARFID = avoidant/restrictive food intake disorder, BN = bulimia nervosa, BED = binge eating disorder, PD = purging disorder, OSFED = other specified feeding or eating disorder, DOI = duration of illness, DASS = Depression Anxiety Stress Scale, MMPI = Minnesota Multiphasic Personality Inventory, BMI = body mass index, CBT = cognitive behavioural therapy, CBT-E = enhanced cognitive behavioural therapy, HC = healthy controls, CASIAN = Clinician Administered Staging Instrument for Anorexia Nervosa, EDNOS = eating disorder not otherwise specified, CIA = Clinical Impairment Assessment questionnaire, EDI = Eating Disorder Inventory.

**Supplementary table 5. Conceptual/review papers data extraction**

| **Author(s), Year, Location** | **Study design** | **Diagnosis** | **Clinical staging** | **Key findings** | **Potential intervention/ implications for treatment** | **JBI Levels of Evidence** |
| --- | --- | --- | --- | --- | --- | --- |
| Beeler et al. 2021 USA | Theoretical review | AN | Two-stage model: Stage 1: Initial Development, Stage 2: Entrenchment | In stage 1, an escalation of dopamine is caused by restriction and exercise, facilitating learning and behavioural plasticity and establishing AN behaviours (eating and activity organised around persistent weight loss). In stage 2, following continued restriction, the dopamine spiral collapses and dopamine becomes impaired. Plasticity decreases, fostering inflexibility and compulsivity and resistance to change. | In stage 1, drugs treatments (e.g., tetrabenazine) can help prevent dopamine escalation and slow disorder onset. In stage 2, dopamine enhancing drugs can promote behavioural flexibility. | Prognosis - 5.c |
| Bodell et al. 2022 Canada | Theoretical review | Binge-type EDs | Three-stage model: Stage 1 - at risk; Stage 2 - recent-onset; Stage 3 - established BED. | Each stage carries distinct patterns of 'wanting' and 'liking' for food and non-food rewards. At-risk individuals show heightened sensitivity to both anticipatory and hedonic aspects of rewards. In recent-onset stage, reward response becomes specifically tuned to food but not to non-food rewards. In stage 3, individuals experience increased wanting for food but reduced liking, perpetuating binging as individuals consume large amounts of food without satisfaction. | Stage-matched treatment targets: for at risk individuals, generalised reward hypersensitivity should be targeted. Approach bias modification or cue-exposure therapy suggested for recent-onset or established binge-type EDs. Options for established binge eating may include mindful eating strategies to re-establish healthy pleasure from food. | Prognosis - 5.c |
| Cosci and Fava 2013 Italy/USA | Systematic review | AN and BN | Four-stage models for AN and BN. Stage 1 - prodromal phase, Stage 2 - acute manifestations, Stage 3 - residual phase, Stage 4 - chronic | Longitudinal and linear progression of AN and BN identified in separate diagnosis-based staging models with 4 stages.  For AN, Stage 1/prodromal phase = uneasiness and fullness after eating, reduced food intake, ‘safe’ foods; Stage 2/acute manifestations = severe restriction, increased activity, denial, increasing preoccupation with body weight/shape/size, social impairment; Stage 3/residual phase; Stage 4/chronic (attenuated or persistent form) = 'extreme emaciation', excessive exercise, ‘general debility’, presence of other psychiatric disorders. For BN, Stage 1/prodromal phase = AN, low self-esteem, depressed mood, anhedonia, anxiety, irritability; Stage 2/acute manifestations = low perceived control over eating, compensatory behaviours; Stage 3/residual phase; Stage 4/chronic (attenuated or persistent form) = 'vicious' cycle of restriction-hunger-binge eating develops. | Stage-matched treatment interventions should be utilised i.e., acute phases requiring more intensive intervention). | Prognosis – 5.a |
| Rizk et al. 2020 France | Systematic review | AN, BN, or EDNOS | Five-stage model: Period 0: preceding AN, Period 1: onset of AN, Period 2: evolution of AN, Period 3: acute phase AN, Period 4: long term outcome | Proposed staging model of problematic physical activity in AN, distinguishing voluntary vs. involuntary components of excessive exercise. Problematic physical activity is prevalent in AN (31-80%) and linked to poor treatment outcomes; excessive exercise may be driven by both conscious control and biological mechanisms. | Problematic physical activity should be assessed in ED treatment plans; classification into voluntary vs. involuntary exercise may improve interventions. | Prognosis – 5.a |
| Treasure et al. 2015 UK, Israel, Australia | Systematic review | AN, BN, and BED | Four-stage model: high-risk markers, prodromal phase, illness chronicity, and treatment resistance. | Staging appears useful for AN, with some evidence for BN and BED, but more longitudinal research is needed. | Early intervention may help prevent progression to severe and enduring EDs; more stage-specific treatments are needed. | Prognosis - 5.a |
| Steinglass et al. 2020 USA | Consensus methods | AN | Proposed a five-stage model for AN: Subsyndromal, Full Syndrome, Persistent Illness, Partial Remission, and Full Remission | Consensus reached on five illness stages (subsyndromal, full syndrome, persistent illness, and partial and full Remission), with broad agreement on restrictive eating as a core feature, that AN progresses through stages, and that AN can be episodic. No or low agreement on duration markers for stages. | Future research should test the validity of the proposed AN staging model. | Prognosis - 5.b |

*Note*. The terms used to describe phases or stages of EDs are mirrored to how those authors described their sample.

AN = anorexia nervosa, BN = bulimia nervosa, BED = binge eating disorder, EDNOS = eating disorder not otherwise specified, ED = eating disorder.

**Supplementary table 6. Qualitative papers.**

| **Author(s), Year, Location** | **Design and methods** | **Diagnosis** | **Clinical staging conceptualisation** | **Key findings** | **Potential intervention/ implications for treatment** | **JBI Levels of Evidence** |
| --- | --- | --- | --- | --- | --- | --- |
| Blackburn et al. 2020 Ireland | Psychoanalytic dynamic narrative approach using psychoanalytically informed interviews, analysed with reflexive narrative analysis. | AN | Longstanding AN, DOI: 8-28, ≥7 years inclusion | Three major themes emerged: “all on our own,” (chaotic childhoods, abuse and mistreatment in early life, continuing into later life); becoming needless (turning inwards and denying basic human needs and joy/pleasure); and “nobody speaks your language.” (clinical teams become an important vessel to meet unmet needs, but often experiencing a lack of empathy and feeling rejected). | Individuals with longstanding AN have unique treatment needs. Addressing early experiences key, as well as a deep understanding of patient desire for understanding and individualised support from clinical teams. | Meaningfulness - 3 |
| Broomfield et al. 2021 Australia | Narrative inquiry using photovoice method and inductive thematic analysis. | AN | Longstanding AN, DOI: ≥7 years | Five themes emerged: transition (distinct turning point to development of longstanding AN, life becomes permeated by AN), trauma (as risk factor), functionality (illness serves a purpose, making recovery difficult; function of AN changes over time), identity (AN may be viewed as part of identity, but some reject personal interconnection), and failure of current models of treatment (ineffective treatments as well as coercive treatment methods). | AN transitions over time and treatment models should adapt to these shifting roles, trauma and social barriers need to be addressed for early intervention, and techniques focusing on identity could help patients detach from AN. | Meaningfulness - 3 |
| Broomfield et al. 2021 Australia | Narrative inquiry using semi-structured interviews analysed under a narrative approach. | AN | Longstanding AN, DOI: ≥7 years | Severe and enduring is a preferred label for this sample of people with AN, and illness duration criteria are deemed appropriate. Concerns expressed about possible negative consequences of labelling and segregating longstanding AN from other AN stages. | Individual impact of labels needs to be considered, especially for longstanding illness definitions. | Meaningfulness - 3 |
| Burke et al. 2024 Australia | Meta-synthesis using reflexive thematic analysis of quotations extracted from 20 studies. | Any ED (excluding pica and rumination disorder) | Longstanding and severe ED, reported mean or range of DOI spanning 6.3-22.4 years. | ‘Lack of Autonomy Loop' develops in longstanding ED, where individuals feel externally controlled first by the ED, and then by treatment systems. Trauma-blind and coercive treatment practices may reinforce ED, as most individuals had experience of multiple traumas. | Shift focus from disease-centred or purely biological progression to person-centred ideas of progression, as longstanding ED is characterised by disturbances in autonomy, with systemic and relational roots. Trauma-informed, relational, and autonomy supportive treatment models need to be established. | Meaningfulness - 1 |
| Cummings et al. 2023 USA | Reflexive thematic analysis of semi-structured interviews. | AN | Severe and enduring AN, (1) continuous history of active AN (excluding temporary periods of weight restoration) for ≥ 7 years after age 18; at least one previous inpatient ED treatment; severe impairment across 2 life domains. | Three themes identified: the all- encompassing nature of SE-AN (subthemes of origins and trajectory of AN, perceived advantages, and known negative consequences), treatment experiences (subthemes of negative experiences and positive experiences), and living with chronic illness (subthemes of living with chronic illness and holding hope). | Effective treatment for longstanding AN should be collaborative and flexible. A harm-reduction approach balancing structure with autonomy could improve engagement and long-term recovery. Better access to specialised care and smoother transitions needed to improve outcomes. | Meaningfulness - 3 |
| Elwyn et al. 2024 Australia | Collaborative autoethnography analysed using reflexive thematic analysis. | AN and OSFED | Longstanding AN, DOI 25 years | Longstanding AN characterised by traumatic experience and is traumatic in itself. Also characterised by unmet treatment needs and painful experiences with healthcare services. For twins, the impact of identity, societal tropes and stigma can interact with ED development and maintenance. | Self-compassion and trauma-informed, identity-affirming care important in longstanding AN recovery. | Meaningfulness - 3 |
| Jack 2000 USA | Modified grounded theory approach using open-ended interviews. | AN | Chronic AN, DOI: ≥10 years | Patient views: AN regulated distance of relationships (closer or more distant); desire for treatment to focus on life issues, not weight; causes = societal pressure, traumatic loss, low self-esteem, genetics, personality, family issues; AN serving as function = avoiding maturity, exerting control, engaging family; looking to future = denial of AN severity, belief that they will recover, hopelessness and recovery not being possible, AN as identity (obstacle to recovery) | Both patients and parents feel ambivalence towards chronic AN and having parental involvement in treatment, an understanding of the balance in family relationships and impact of past treatments is needed. | Meaningfulness - 3 |
| Kiely et al. 2025 Australia | Meta-ethnographic meta-synthesis of qualitative studies. | AN | SE-AN (DOI > 3 years) | A new theme, 'walking on a knife’s edge, caught between worlds' suggests that SE-AN contains tensions within the dialectic of living, fighting, surviving, and dying. Incomplete clinical knowledge on SE-AN results in failures by treatment systems. Terminal AN concept perceived as a risk to hope building, which is critical to SE-AN recovery. | Stage-adapted care is needed. For SE-AN treatment, key treatment needs are: building trust and hope, respecting autonomy, offering trauma-informed, compassionate, flexible care, and care that is tailored to individual identity and recovery goals. | Meaningfulness - 1 |
| Kiely et al. 2024 Australia | Case study analysed using interpretative phenomenological analysis of published autobiographical material. | AN | SE-AN, DOI 18 years | SE-AN is self-reinforcing and substitutes the individual's identity, resulting in a 'disappearing self'. A dialectical dilemma emerges where AN is both safe, and unsafe, resulting in a 'stalemate'. However, finding life-meaning and purpose can drive new hope. | SE-AN not defined solely by duration or severity, but also by self/agency/identity disturbances. Suggestions to remove underweight criterion to SEED, and including ‘global impoverishment’ and self and identity to defining features of this stage. | Meaningfulness - 3 |
| Kiely et al. 2023 Australia | Meta-ethnographic meta-synthesis of qualitative studies. | AN or atypical AN | Severe and enduring AN, DOI:> 3 years DOI | Severe and enduring AN from a treatment seeking perspective can be conceptualised as a relational, existential disorder of the self. Individuals may become trapped in AN, losing sense of self, quality of life, control, and hope. A four-phase recursive model is proposed, metaphorically illustrated as a spider and its web (symbolising AN entrapment). | Personalised, compassionate, and recovery-oriented treatment approaches needed for longstanding AN that address identity, shame, and agency rather than weight restoration alone. Flexible, patient-centred care including self-admission and peer support may empower individuals and improve engagement. | Meaningfulness - 1 |
| Koskina et al. 2019 UK | Case study with in-depth description of treatment process. | AN | Early stage AN, DOI: ≤ 3 years | Significant improvement in ED symptoms, BMI and psychological distress over the course of treatment were observed and sustained to extended follow-up and patient's transition to university. | Incorporation of identity exploration and development in the treatment of early-stage AN is acceptable and effective | Meaningfulness - 3 |
| Lindstedt et al. 2025  Sweden | Thematic analysis of semi-structured interviews. | AN | N/A – high/low polygenic risk sample | Individuals with high polygenic risk for AN showed a more persistent and internally driven illness course that felt intertwined with their identity. Those with low polygenic risk described more environmentally triggered, episodic illness seen as a deviation from their life trajectory, and had greater capacity to distance themselves from AN with recovery. | Genetic risk may meaningfully shape clinical trajectories and could help identify individuals at risk for more severe or enduring forms of the disorder. | Meaningfulness - 3b |
| Meneguzzo et al. 2025  Italy | Open‑ended questionnaire analysed via reflexive thematic analysis and longitudinal comparison of narrative change | AN, BN, BED | Early stage ED, DOI: ≤3 years  Enduring ED, DOI: >3 years | Early stage EDs showed more variability and were more likely to experiment with reducing activity, often with ambivalence. Those with longer DOI maintained rigid, established activity patterns and described movement as habitual, identity‑linked, and resistant to modification. | Early intervention is crucial, as greater flexibility demonstrated  in shorter DOI cases versus identity entanglement related to physical activity in longer DOI cases. | Meaningfulness - 3b |
| Robinson et al. 2024 UK | Thematic analysis of semi-structured interviews. | BN | SEED-BN/SE-BN (≥5 years) | Participants described certain enduring psychological and physical problems with their BN, such as dental and gastrointestinal symptoms. Many expressed a loss of faith in therapy, and a desire for alternate treatment approaches. Half were in employment but experienced isolation and difficulties with interpersonal relationships. | Treatment should prioritise improving quality of life and social and occupational functioning, in addition to psychological, medical, and dietetic approaches. | Meaningfulness - 3 |
| Robinson et al. 2015 UK | Thematic analysis of semi-structured interviews. | AN | Severe and enduring ED-AN, DOI: ≥20 years. | Patients with SEED-AN described psychological resilience but also chronic hopelessness; low motivation for recovery was common. | SEED-AN patients may benefit from psychosocial interventions focusing on quality of life rather than full recovery. | Meaningfulness - 3 |
| Skoufalos et al. 2011 USA | Psychological phenomenological analysis of semi-structured interviews and focus group. | BN and subclinical BN | Subclinical and full threshold BN | BN and subclinical BN share core factors such as fear of rejection, body image concerns, and sociotropy but their organisation and intensity differ. BN = deeper sense of chronic loneliness, stronger emotional distress, and more entrenched binge-purge cycles as coping mechanism. SCBN = similar, but less severe or frequent behaviour engagement. | BN and subclinical BN exist on a spectrum, more nuanced diagnostic criteria is needed to consider severity, psychological distress, and behavioural patterns opposed to categorical definitions of illness. | Meaningfulness - 3 |
| Stockford et al. 2018 UK | Semi-structured interviews analysed using IPA to explore psychological meaning of AN symptoms. | AN | Severe and enduring AN, DOI between 14 and 28 years, having a variety of interventions but no sustained progress. | Participants viewed AN as functional and as serving psychological difficulties. A lack of early intervention was prominent, and treatment experiences were often negative and related to the maintenance of AN. | SE-AN patients may benefit from psychological interventions addressing identity and self-worth rather than traditional weight-focused treatments. | Meaningfulness - 3 |
| Wojtkowiak et al. 2023 Netherlands | Thematic analysis of semi-structured interviews and focus group. | No specific diagnoses | Severe and enduring EDs | Three main themes identified. The first focused on the uncertainty around dying in SEEDs, with desire to maintain hope for patient but also considering that their patient may not recover. The second focused on dilemmas in treating SEEDs, namely, what is treatment resistance, and what is palliative care, as there is no consistent meaning across providers. The last theme focused on suicidal ideation or intent, resulting from patient's weariness and sense that life is not worth living. However, suicidal intent can be treated. | Clarity and discussion around end-of-life issues is needed for mental healthcare providers working with SEEDs. | Meaningfulness - 3 |

*Note*. The terms used to describe phases or stages of EDs are mirrored to how those authors described their sample.

AN = anorexia nervosa, ED = eating disorder, OSFED = other specified feeding or eating disorder, SE-AN = severe and enduring anorexia nervosa, SEED = severe and enduring eating disorder, SEED-AN = severe and enduring eating disorder – anorexia nervosa, SEED-BN = severe and enduring eating disorder – bulimia nervosa, BN = bulimia nervosa, SCBN = subclinical bulimia nervosa, IPA = interpretative phenomenological analysis.
